# Clinical and Radiological Dissociation in Massive Barium Aspiration

**DOI:** 10.7759/cureus.12557

**Published:** 2021-01-07

**Authors:** Ramakanth Pata, Tsering Dolkar, Meet J Patel, Nway Nway, Htun M Aung

**Affiliations:** 1 Internal Medicine, Interfaith Medical Center, Brooklyn, USA

**Keywords:** barium aspiration

## Abstract

Barium studies are commonly used to rule out gastrointestinal (GI) pathologies and sometimes they are associated with complications such as barium aspiration with heterogeneity in clinical features ranging from mild to severe symptoms. We present a case of large volume barium aspiration in a 73-year-old male with past medical history of dysphagia diagnosed with diffuse esophageal spasm. Barium is an inert material commonly used for GI tract study. Although complications associated with barium studies are rare, aspiration of barium can have dramatic findings resulting in mild to severe symptoms. Clinically patient had very minimal symptoms but radiographic studies appeared dramatic. Therefore, a clinical and radiographic paradox must be kept in mind when evaluating patients and reviewing large volume barium aspiration imaging. Our case remained asymptomatic and had no respiratory complaints, nor did he develop any respiratory distress post barium aspiration.

## Introduction

Barium is an inert material commonly used for gastrointestinal (GI) tract study. We present a case of large volume barium aspiration in a 73-year-old male with past medical history of dysphagia diagnosed with diffuse esophageal spasm. Although complications associated with barium studies are rare, aspiration of barium can have dramatic findings resulting in mild to severe symptoms. Clinically patient had very minimal symptoms but radiographic studies appeared dramatic.

## Case presentation

**Figure 1:**
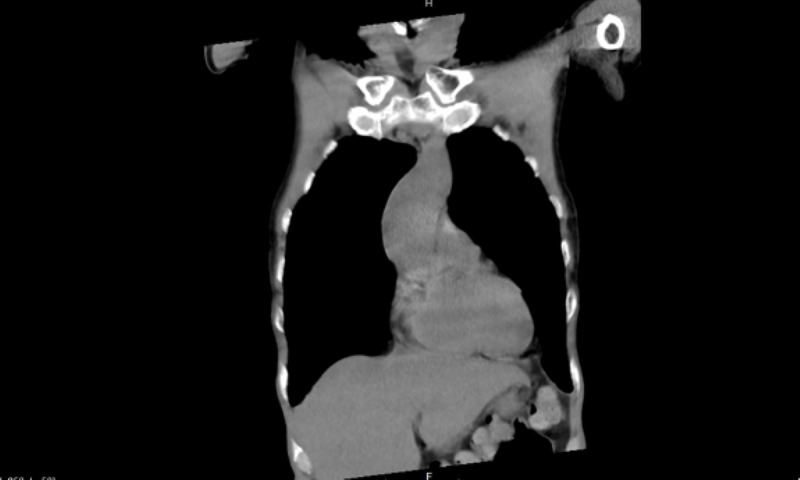
CT chest without contrast revealed fluid-filled dilated esophagus, which was suboptimally evaluated due to lack of contrast. The patient also had left lower lobe consolidation consistent with pneumonia.

**Figure 2:**
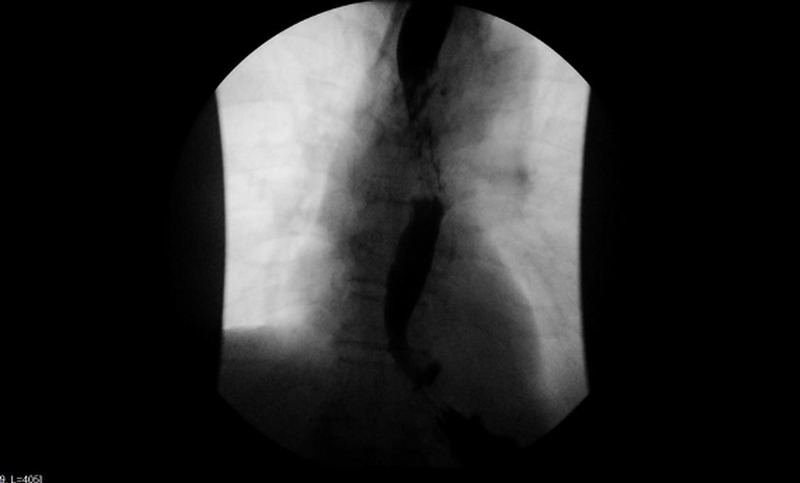
A barium study was done, which revealed diffuse esophageal spasm and akinesis of the mid-thoracic esophagus.

**Figure 3:**
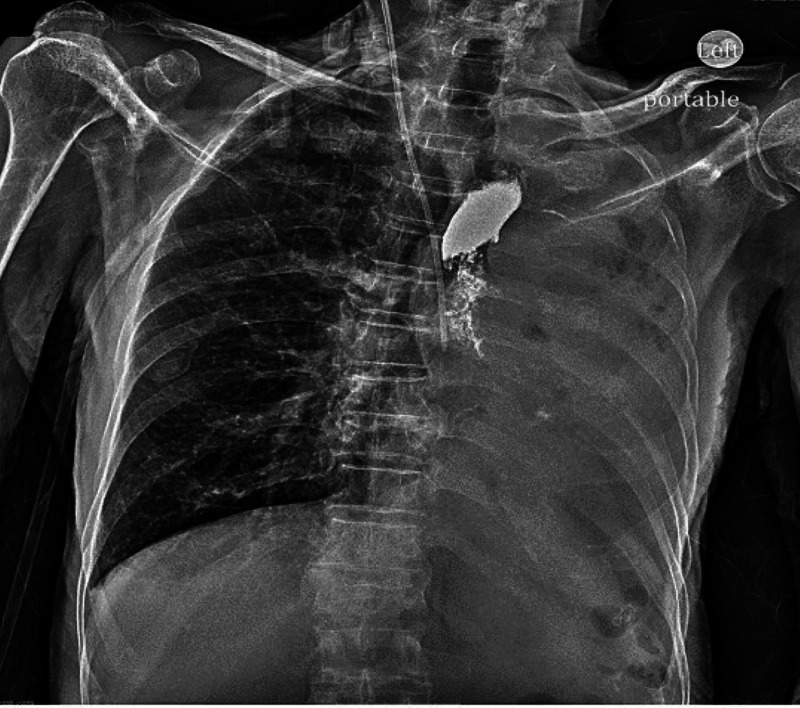
Chest X-ray revealed interval development of complete opacification of left hemithorax and retained barium in the left upper chest.

**Figure 4:**
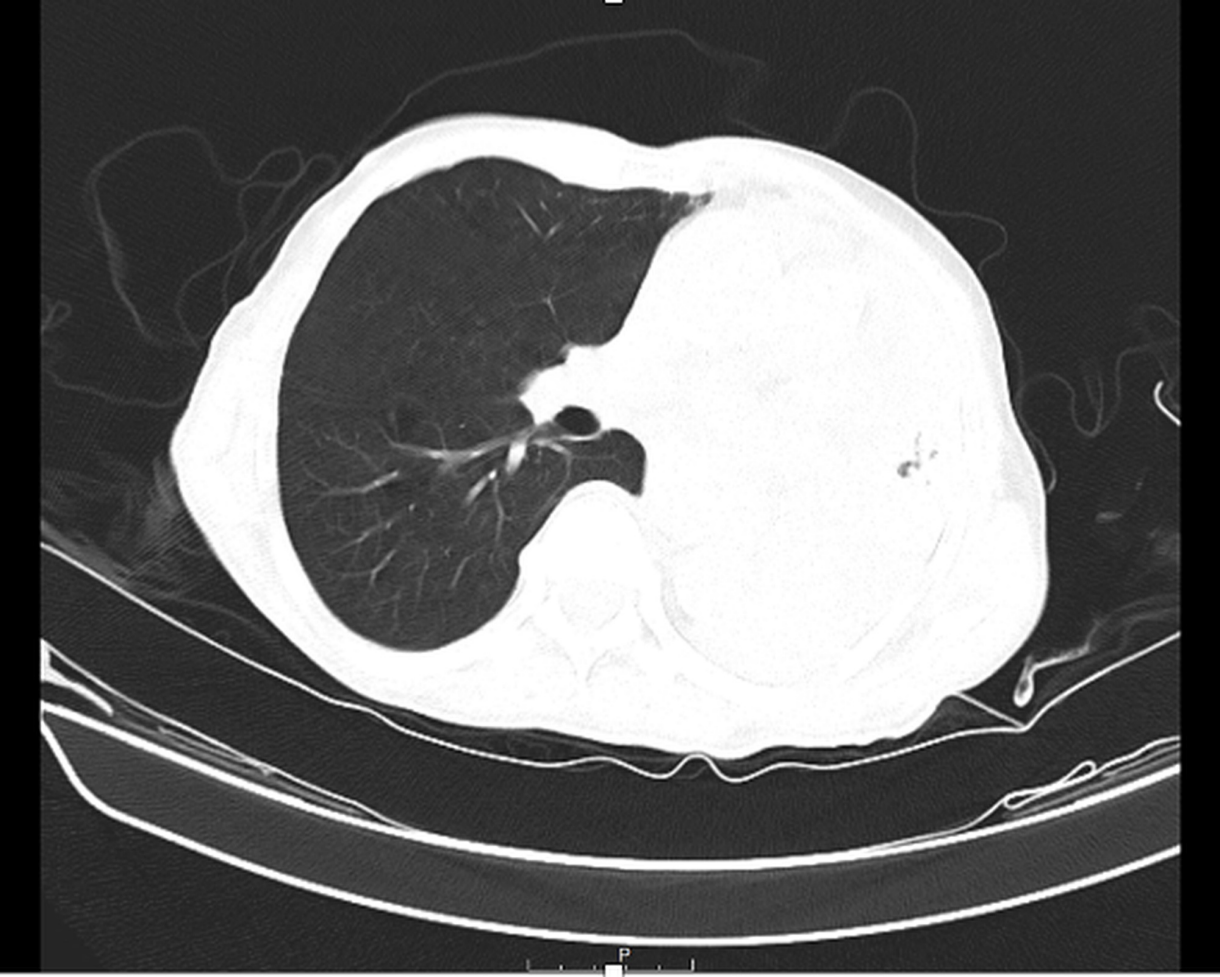
CT chest revealed retained barium within the upper to the mid-thoracic esophagus. Complete left lung atelectasis was noted, likely related to the obstructing filling defect within the left main bronchus.

**Figure 5:**
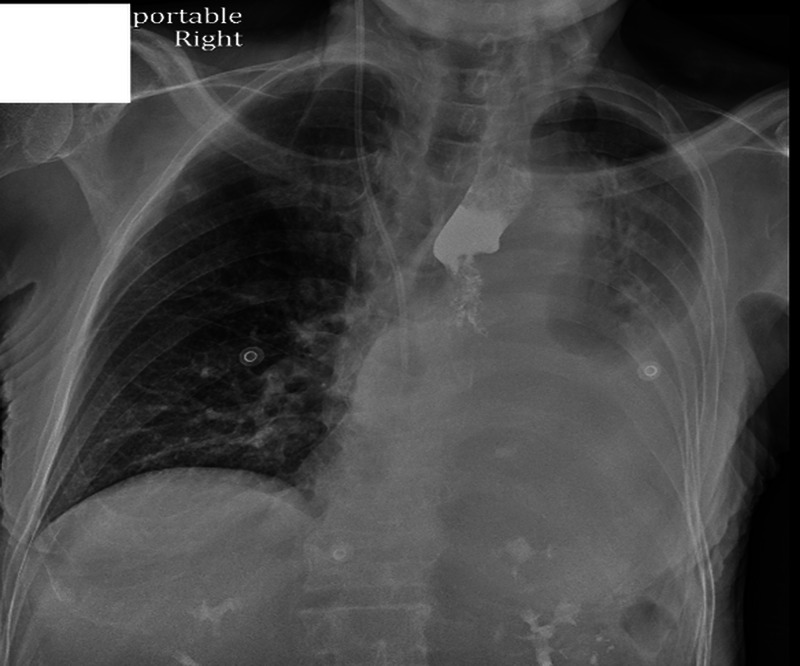
Subsequently, a chest x-ray showed persistently retained contrast within the esophagus and improved left lobar airspace disease with moderate to large pleural effusion.

A 73-year-old male presented to emergency department due to presyncope with an episode of loss of consciousness for three minutes. He also complained of productive cough with whitish sputum. Vital signs were normal and physical examination was benign. Chest X-ray (CXR) showed evidence of consolidation, which was followed by a computed tomography (CT) chest that revealed a dilated esophagus (Figure 1). Barium swallow was done for optimal evaluation of dilated esophagus (Figure 2). Post-procedure CXR revealed interval development of complete opacification of left hemithorax and retained barium in the left upper chest (Figure 3). CT chest was followed, which revealed retained barium within the upper to the mid-thoracic esophagus. Complete left lung atelectasis was noted, likely related to the obstructing filling defect within the left main bronchus (Figure 4). Subsequently, the CXR showed persistently retained contrast within the esophagus and improved left lobar airspace disease with moderate to large pleural effusion (Figure 5). However, bedside sonogram showed predominantly consolidation. The patient had no symptoms and was clinically stable to be discharged. He was advised to follow up as an outpatient at the chest clinic in six weeks.

## Discussion

Barium studies are regularly performed to rule out GI lumen pathologies. Despite the risk of barium aspiration, which is present, the complications are rare [1,2]. We present a case of large volume barium aspiration which was clinically asymptomatic with dramatic radiographic findings. In most cases, patients remain largely asymptomatic despite the paradoxical radiographic findings [3]. However, mechanical obstruction leading to atelectasis as well as uncommon acute inflammatory reactions have been reported [3-5].

There are few articles published reporting mild cases of large volume barium aspiration [1]. Most patients do not develop severe symptoms as seen on the imaging, which is alarming. Our case remained asymptomatic and neither had any respiratory complaints nor did he develop any respiratory distress post barium aspiration. CT chest revealed complete left lung atelectasis, which subsequently improved within days, revealing moderate pleural effusion. Although there was a pleural effusion on the CXR, a bedside sonogram of the lung revealed a predominant consolidation pattern. The patient was kept under observation. A 6-minute walk test was carried out and no desaturation or symptoms were noted. Steroids or antibiotics were not used and bronchoscopy was not performed due to the risk of spreading the contrast to unaffected areas [4]. The patient was asked to follow up in the pulmonary clinic six weeks post-discharge.

We prefer to classify patients based on radiographic, physiologic, and clinical parameters: positive radiographic findings and no change in physiological findings; positive radiographic findings and altered ventilation/perfusion (V/Q) ratio [6]; positive radiographic findings with shunt effect [7]; and positive radiographic findings with inflammatory response [8]. Most patients are completely asymptomatic after aspiration and treatment remains observation [2]. Aspiration pneumonia should be suspected whenever a patient complains of dysphagia and radiographic evidence of consolidation. Nevertheless, the risk of aspirations and complications remains high in patients with dysphagia or the elderly [1,9]. Treatment strategies vary depending on the underlying baseline comorbidities in patients with severe respiratory complications, although bronchoscopy and suctioning have been tried [10]. Furthermore, if gastric contents are aspirated along with barium, antibiotics have been used [11].

Most patients appear to recover completely one-year post aspiration. High-resolution CT has reported subtle evidence of early fibrosis even one-year post aspiration, although limited data is available on patient outcome years after aspiration [3]. The clinical significance of such findings remains unknown.

## Conclusions

Although barium aspiration has dramatic radiographic findings, most patients are asymptomatic. Therefore, a clinical and radiographic paradox must be kept in mind when evaluating the patients and reviewing large volume barium aspiration imaging. We recommend longer follow-ups to check for delayed developments post barium aspiration.
